# *Notes from the Field:* E-Cigarette–Associated Cases Reported to Poison Centers — United States, April 1, 2022–March 31, 2023

**DOI:** 10.15585/mmwr.mm7225a5

**Published:** 2023-06-23

**Authors:** Nicole A. Tashakkori, Brian L. Rostron, Carol H. Christensen, Karen A. Cullen

**Affiliations:** 1 Center for Tobacco Products, Food and Drug Administration, Silver Spring, Maryland.

E-cigarette–associated cases reported to U.S. poison centers have fluctuated during the past decade, increasing during 2010–2014, and then decreasing during 2015–2017 (*1*). During 2017–2018, the number of e-cigarette exposure cases increased by 25% (from 2,320 to 2,901), and in 2018 nearly two thirds (63.3%) of cases occurred among children aged <5 years (*1*). To understand the number and characteristics of e-cigarette exposure cases in the United States, the Food and Drug Administration (FDA) analyzed National Poison Data System (NPDS) data* from the most recently available 12-month period (April 1, 2022–March 31, 2023). NPDS is maintained by U.S. poison centers. FDA’s analyses report a further increase in the number of e-cigarette exposure cases, particularly among children aged <5 years.

NPDS is a repository of cases reported to U.S. poison centers that are recorded by specially trained and certified health care professionals (*2*). Information on exposure cases (reports or reported incidents by persons who contact poison centers regarding an exposure to a substance) in NPDS is recorded based on generic codes (a required general identification code for a substance or group of products) and product codes (product-specific codes, often by brand; these are not required upon case intake). Cases involving e-cigarettes were identified using generic codes; brands were identified using product codes.^†^ E-cigarette exposure cases were defined as an exposure to e-cigarettes or e-liquids and were examined by age group, exposure route, level of care provided, medical outcome, and product brand. This study was determined as exempt by the FDA Institutional Review Board for Human Subject Protection.^§^

During April 1, 2022–March 31, 2023, a total of 7,043 e-cigarette exposure cases were reported (Table), representing a 32% increase, from 476 in April 2022 to 630 in March 2023. Among all exposures, 6,074 (87.8%) occurred among children aged <5 years. Inhalation or nasal (4,298; 61.0%) and ingestion or oral (2,818; 40.0%) exposure routes were most common. Overall, 43 (0.6%) e-cigarette exposure cases resulted in hospital admission, and 582 (8.3%) required treatment at a health care facility. A major effect^¶^ was experienced in 12 (0.2%) exposure cases and a moderate effect in 133 (1.9%) cases. One reported case resulted in death (a suspected death by suicide of a person ≥18 years). Approximately one half of reported cases resulted in either a minor effect (27.2%) or no reported effect (19.8%); 50.9% of cases were not followed.** Among 342 (4.9%) cases with brand information, the most commonly reported brand was Elf Bar (60.8%), a disposable e-cigarette available in a variety of flavors; monthly cases involving Elf Bar increased from two in April 2022 to 36 in March 2023. More than 90% of Elf Bar exposures were among children aged <5 years.

**TABLE:** Characteristics of poisoning exposures involving e-cigarettes (N = 7,043)* — United States, April 1, 2022–March 31, 2023

Characteristic	No. (%)
**Age group, yrs^†^**	
<5	6,074 (87.8)
5–11	206 (3.0)
12–17	153 (2.2)
18–24	198 (2.9)
≥25	288 (4.2)
**Exposure route^§^**	
Inhalation or nasal	4,298 (61.0)
Ingestion	2,818 (40.0)
Dermal	245 (3.5)
Ocular	67 (1.0)
Other^¶^	39 (0.6)
**Level of care at health care facility**	
Not referred	6,113 (86.8)
Refused referral or did not arrive	100 (1.4)
Lost to follow-up or left against medical advice	205 (2.9)
Treated, evaluated, and released	582 (8.3)
Admitted to a hospital**	43 (0.6)
**Medical outcome**	
Not followed ^††^	3,584 (50.9)
No effect	1,398 (19.8)
Minor effect	1,915 (27.2)
Moderate effect	133 (1.9)
Major effect	12 (0.2)
Death	1 (0.01)
**Brand** ^§§^	
No brand reported	6,701 (95.1)
Brand reported	342 (4.9)
Elf Bar	208 (60.8)
JUUL	55 (16.1)
Vuse	31 (9.1)
Pop Vape^¶¶^	20 (5.8)
Puff Bar	14 (4.1)
Other brand***	14 (4.1)

* Cases involving exposure to more than one substance were excluded.

^†^ Missing or incomplete data are excluded in the percentage values for age. Data are considered missing or incomplete when no information is provided for the variable or when listed as unknown persons aged ≤19 years, or unknown persons aged 20–29 years. Two persons listed as being aged 30–39 or 50–59 years are categorized as aged ≥25 years. Data are missing or incomplete for 124 persons.

^§^ More than one exposure route was possible for each case; thus, percentages might not sum to 100%.

^¶^ Includes less commonly reported routes of exposure, such as aspiration (with ingestion), optic, parenteral, rectal, vaginal, and unknown routes.

** Patients are categorized as having been admitted to a hospital when coded as being admitted to a critical care unit, noncritical care unit, or psychiatric facility.

^††^ Data are considered not followed when coded as 1) not followed, judged as nontoxic exposure (clinical effects not expected); 2) not followed, minimal clinical effects possible (no more than minor effects possible); and 3) unable to follow, judged as a potentially toxic exposure.

^§§^ The percentages reported by brand represent the number of exposures for each brand out of the total number of exposures where brand was reported.

^¶¶^ Data for Pop Vape were not available until April 30, 2022.

*** “Other brand” includes five cases reported for exposure to Myle Vapor, three for Bidi Stick, two for 7 Daze Pods, two for Aquabar, one for SMPO, and one for Suorin. No cases were reported during this period for Green Smoke, Bo Caps, Crossbar, or MarkTen.

NPDS relies on voluntary reporting of poisoning exposure cases; thus, the number of cases is likely underreported (*3*). In addition, because product codes are not required, only a small proportion of e-cigarette exposure cases included information on the brand associated with the exposure.

The number of reported U.S. e-cigarette exposure cases during this 12-month period is approximately double the number reported in 2018 (*1*). Most of the cases were among children aged <5 years. Among the 5% of cases for which brand was available, Elf Bar, for which sales in the United States have recently increased (*4*), was reported more often than all the other reported brands combined, with nearly all Elf Bar cases occurring among children aged <5 years.

Continued surveillance is critical to guiding efforts to prevent poisoning exposure associated with e-cigarettes, particularly among young children. Health care providers; the public health community; e-cigarette manufacturers, distributors, sellers, and marketers; and the public should be aware that e-cigarettes have the potential to cause poisoning exposure and are a continuing public health concern (*5*). Adult e-cigarette users should store their e-cigarettes and e-liquids safely to prevent access by young children.

## Footnotes

*
https://poisoncenters.org/

† Product codes are only available for 16 e-cigarette brands in NPDS.

§ This study was determined as exempt by the FDA Institutional Review Board for Human Subject Protection because data were previously collected and did not contain personally identifiable information.

¶ On the basis of definitions provided by NPDS, patients experiencing a minor effect exhibit some signs and symptoms from the exposure, which would usually resolve rapidly, such as mild, self-limited gastrointestinal symptoms, without dehydration or transient cough. Persons experiencing a moderate effect exhibit more pronounced and prolonged signs and symptoms for which some form of treatment would be indicated, such as a high fever, disorientation, or gastrointestinal symptoms causing dehydration. Major effects from exposure are life-threatening or might result in severe signs and symptoms (e.g., repeated seizures, cardiac arrest, or respiratory arrest), severe disability, or disfigurement.

** Data are considered not followed when coded as 1) not followed, judged as nontoxic exposure (clinical effects not expected); 2) not followed, minimal clinical effects possible (no more than minor effects possible); and 3) unable to follow, judged as a potentially toxic exposure.
